# Supplementary material for: deSALT: fast and accurate long transcriptomic read alignment with de Bruijn graph-based index
Source: Genome Biol. 2019 Dec 16;20:274. doi: 10.1186/s13059-019-1895-9 (PMC6913027; doi:10.1186/s13059-019-1895-9)
Supplement: Supplementary file 2 — Additional file 2. Review history. [file 13059_2019_1895_MOESM2_ESM.docx]

Review History

**First round of review**

**Reviewer 1**

**Were you able to assess all statistics in the manuscript, including the appropriateness of statistical tests used?**

No

**Were you able to directly test the methods?**

Yes

**Comments to author:**

\### Summary

The manuscript presents deSALT, a long read transcriptomic aligner. deSALT is a two-pass aligner that attempts, in the first pass, to find a skeleton (chain) of match blocks of each read and to use those skeletons to find the exons present in the read set. In the second pass, deSALT uses the identified exons along the match blocks of a given read to extend and refine its mapping and alignment.

The manuscript is well presented, the experiments are cover a lot of ground, methods are well explained, and the installation and use of the tool are very simple and hassle-free. The overall performance of deSALT surpasses state-of-the-art transcriptomic aligners. In the specific cases presented (e.g. small exons or tricky splice sites), deSALT seems to exceptionally excel compared to other tested tools.

\### Major Comments

\#### Results

- Since deSALT has an annotation-aware mode that is assessed, minimap2 latest version (v2.17) --junc-bed option should also be tested in order to have a complete comparison.

\#### Tests

While the tested criteria show that deSALT has great performance, they need to be augmented to test for potential pitfalls:

- I highly recommend including GraphMap2 (http://doi.org/10.1101/720458) to your results. The original GraphMap (https://doi.org/10.1038/ncomms11307) was published in 2016 and its successor's manuscript claims higher accuracy (albeit slower time) than Minimap2.

- For all time result presented, please indicate whether it is CPU (user time) or wall clock (real) time. When presenting results for multi-threaded tests, please include both.

- How does deSALT perform on reads coming from lowly expressed isoforms that are part of a gene with many other isoforms? Perhaps adding a set of genes with variable isoform level expression would address this.

- How does deSALT perform on pairs of genes that overlap genomically but are on opposite strands? Would the potential for overlapping exons cause problems for alignment accuracy? Additional tests highlighting this issue are needed.

- What is the memory use of all the methods?

- What are the memory/time footprint of the indexing stage? I tested deSALT indexing on GRCh38 and it took over 70Gb and over two hours.

- Since GMAP and deSALT prebuild their indices, when comparing to Minimap2 its index should also be prebuilt. The command provided does not seem to indicate that.

\#### Methods

- It is not clear how the RdBG index is queried with l-mers given that the index was constructed with k-mers of size k>l. What is the auxiliary data structured that allows that?

- "Implementation of the simulation benchmark":

- How was the coverage calculated? Since we are dealing with transcriptome here the definition of coverage is not as clear it is in the case of genomics.

- The second step is not clear. Specifically the second sentence: "the transcript sequences were generated according to all its isoforms"

- How was the specific genes in each of the three gene sets selected? Were they randomly selected? If there is no strong reason then I think that all protein coding genes should be included in their respective gene sets?

- Where are GMAP results in Figure 2 for ONT 1D data?

- "3.2 Alignment of the read part against the LSRS":

- What constitutes "large deletions" in the CIGAR?

- "2.2 Exon refinement":

- How is the accepter/donor matrices derived?

\### Minor Comments

- For ease of read, I suggest converting all time into minutes and all memory to GB.

- "Results on simulated datasets":

- What is meant by "the Exon% statistics (about 64%-90%) were affected to some extent"? What is the percentage range referring to?

- P4L9: "most of them are relatively low speed" --> "most of them have relatively slow speed"

Collapse

- (not required but highly recommended) Please consider adding the deSALT to bioconda packages for making it more accessible for general audience.

\### Recommendation

**Reviewer 2**

**Were you able to assess all statistics in the manuscript, including the appropriateness of statistical tests used?**

Yes

**Were you able to directly test the methods?**

Yes

**Comments to author:**

In this manuscript, Liu et al. introduce deSALT, a new method of aligning long-read transcriptomic data against a reference genome using a deBruijn graph-based index. This approach builds upon some of the previous work of some of the authors (the RdBG index, as introduced in deBGA), but makes entirely different use of this index and also adds many novel concepts specifically related to the alignment of long spliced (i.e. transcriptomic) reads. deSALT looks like a very promising new approach, and the manuscript provides a detailed and precise description of the method. The assessment is generally convincing. However, there are some places where extra information could be provided or the manuscript might be improved.

* It seems that an assessment of the memory requirements are not given anywhere within the current manuscript. Along with accuracy and alignment speed, the memory usage of the proposed approach is a critical metric to report, as it informs in which contexts the tool might be used (e.g. on cluster nodes with limited memory, or in memory-limited cloud instances).

* The authors state that PBSim is used for generating all of the simulated data, where different types of simulated samples are created by changing the characteristic read lengths and error rates. However, the error characteristics of PacBio and ONT data differ in many important ways apart from simply the rate of error. Thus, it is unlikely that reads generated by PBSim with a high error rate and a longer mean read length are truly representative of how nanopore reads are likely to look. I'd recommend that the authors use a nanopore-specific simulator to benchmark on simulated ONT data --- there are a few options available like NanoSim or DeepSimulator.

* When performing an assessment on experimental samples, it would be useful to look at both cDNA and direct RNA-based nanopore sequencing. Though similar, the characteristics of these protocols are not identical, and it seems important to know how the different methods perform on both types of data (though I have no a priori reason to believe that deSALT's improvements won't hold equally-well in both situations).

* Given that deSALT builds a secondary k'-mer index (where k' < k) index over the RdBG index, it was not particularly clear what the benefit or motivation was for using a graph-based index as opposed to simply building a k'-mer index over the input reference. When k' = k (as in deBGA), there is a benefit that any U-MEM is unique within the dBG, however in the case k' < k, only U-MEMS of length at least k are guaranteed to be unique in the dBG. Is it the case the performing the initial seeding over the dBG as opposed to the reference improves efficiency by reducing redundancy during seeding? Is this still the case even though k' < k? The authors state that altering the value of k has little effect on the results or the runtime, but what is the effect of changing the value of k'. This is a crucial parameter for this algorithm, and so some assessment of its effect would be useful to have.

* It would be useful if the authors provide some technical detail about the k'-mer index they build over the RdBG. Is this simply a hash table with a list of the positions of each k'-mer in the RdBG? How are the occurrences encoded? What is the size of this data structure and how does it depend on k'? Are the k'-mers canonicalized?

minor comments

-------------------

* There were some places where the phrasing within the manuscript was awkward or value-laden (e.g. "deSALT also obviously outperformed other state-of-the-art aligners"). I would recommend removing such unnecessary adverbs, or adjectives, as they don't really contribute to the point being made.

* The formatting of the paper was a bit strange in places. Paragraphs would begin with "#)" followed by a paragraph heading, then a newline before the paragraph begins. I'd suggest the authors make use of more traditional paragraph or subsection headings.

* It seems that, if one were to forgo the skeleton creation step, then deSALT may also be appropriate as a DNA-seq aligner for long read data. Have the authors done any testing of the method for this purpose? Can deSALT be used to perform un-spliced alignment of long-read data?

**Authors Response**

**Point-by-point responses to the reviewers’ comments:**

---------------------------------------------------------------------------------------------------------------

Response to reviewer #1

---------------------------------------------------------------------------------------------------------------

Reviewer #1: ### Summary

The manuscript presents deSALT, a long read transcriptomic aligner. deSALT is a two-pass aligner that attempts, in the first pass, to find a skeleton (chain) of match blocks of each read and to use those skeletons to find the exons present in the read set. In the second pass, deSALT uses the identified exons along the match blocks of a given read to extend and refine its mapping and alignment.

The manuscript is well presented, the experiments are cover a lot of ground, methods are well explained, and the installation and use of the tool are very simple and hassle-free. The overall performance of deSALT surpasses state-of-the-art transcriptomic aligners. In the specific cases presented (e.g. small exons or tricky splice sites), deSALT seems to exceptionally excel compared to other tested tools.

*--Response:*

*Thank you very much for reviewing our manuscript. We appreciate all the reviewers’ comments and suggestions. We have supplied new benchmark results and revised the manuscript according to the reviewer’s comments. A point-by-point response has been made according to the comments as follows.*

\### Major Comments

\#### Results

- Since deSALT has an annotation-aware mode that is assessed, minimap2 latest version (v2.17) --junc-bed option should also be tested in order to have a complete comparison.

*--Response:*

*We have added the results of Minimap2 with --junc-bed option in the benchmark. Please refer to Figures 2-5 and Supplementary Tables 2-6 and 8-10 for the results. The results suggest that both of deSALT and Minimap2 can improve their alignment with the help of gene annotations, especially for error-prone reads. Moreover, the yields of deSALT and Minimap2 are closer to each other with gene annotations. We have described this issue in the current version of the manuscript as follows:*

*“deSALT can further improve the alignment of error-prone reads with gene annotations. We used Ensembl gene annotations as input to benchmark the alignments of deSALT and Minimap2. The results (Figure 2A and Supplementary Table 2) demonstrate that both of deSALT and Minimap2 had better yields with the help of gene annotations, especially for the alignment of the error-prone ONT 1D reads. Moreover, the difference between the yields of the two aligners is also smaller.” (line 39, page 9 of main text)*

\#### Tests

While the tested criteria show that deSALT has great performance, they need to be augmented to test for potential pitfalls:

- I highly recommend including GraphMap2 (http://doi.org/10.1101/720458) to your results. The original GraphMap (https://doi.org/10.1038/ncomms11307) was published in 2016 and its successor's manuscript claims higher accuracy (albeit slower time) than Minimap2.

*--Response:*

*Thank you very much for this suggestion. We have added GraphMap2 in the benchmark. Please refer to Figures 2-5 and Supplementary Tables 2-6 and 8-10 for the results. Overall, the yields of GraphMap2 can be comparable to that of Minimap2, but still lower than that of deSALT. Moreover, as the reviewer mentioned, the speed of GraphMap2 is slower. Meanwhile, we observed that the memory footprint of GraphMap2 increased with the size of the dataset, and it used more memory than other benchmarked aligners. It is also worth noting that, GraphMap2 unaligned most of the reads of the ONT direct RNA dataset and raised a “segmentation fault” for the PacBio dataset during the benchmark, so we did not show its result on these two datasets.*

*We have updated current manuscript as follows:*

*“deSALT and three state-of-the-art approaches, Minimap2, GraphMap2 and GMAP, were applied to all 60 datasets for comparison. In the benchmark, the indexes of all the aligners were pre-built. Five metrics (i.e., Base%, Exon%, Read80%, Read100%, and #Bases/m) were used to assess the sensitivity, accuracy and performance of the aligners (refer to the Methods section for the definitions). The results of the simulated datasets are provided in Figure 2 and Supplementary Tables 2-8. Mainly, five observations were made.” (line 1, page 7 of main text)*

*“It is also worth noting that both of GraphMap2 and Minimap2 outperformed GMAP. Moreover, GraphMap2 and Minimap2 outperformed each other on various datasets and various metrics, and they overall had comparable yields.” (line 26, page 7 of main text)*

*“It is observed that the memory footprints of GraphMap2 increased with the number of input reads, and higher than that of all the other benchmarked aligners.” (line 13, page 8 of main text)*

*“It is also worth noting that we only showed the results of GraphMap2 on the ONT cDNA dataset, since it unaligned most of the reads of the ONT direct RNA dataset and raised a “segmentation fault” for the PacBio dataset during benchmarking.” (line 26, page 10 of main text)*

- For all time result presented, please indicate whether it is CPU (user time) or wall clock (real) time. When presenting results for multi-threaded tests, please include both.

*--Response:*

*Thank you very much for pointing this out. We have added both CPU time and wall clock time for all the time result presented. Due to the limited space, we only showed wall clock time in Figure 2B, and both of CPU time and wall clock in Supplementary Tables 8 and 9.*

*We have mentioned in the current version of the manuscript as follows:*

*“#Bases/m: the number of bases aligned per minute, which depicts the alignment speed and is computed by N_base⁄T_aln , where N_base is the total number of bases in the dataset and T_aln is the wall clock time.” (line 53, page 17 of main text)*

*“Also refer to Supplementary Table 8 for a more comprehensive assessment of the alignment speeds and memory footprints in various numbers of CPU threads (1, 4, 8, and 16 CPU threads).” (line 56, page 25 of main text)*

*“The time costs with 24 threads were also assessed (both wall clock time and CPU time, Supplementary Table 9), and it suggests that deSALT and Minimap2 has similar speed and both of them are faster than GMAP and GraphMap2.” (line 44, page 10 of main text)*

- How does deSALT perform on reads coming from lowly expressed isoforms that are part of a gene with many other isoforms? Perhaps adding a set of genes with variable isoform level expression would address this.

*--Response:*

*deSALT is able to handle the reads from genes with many isoforms. We have added a series of simulated datasets with various sequencing error rates in the benchmark to address this issue. We composed these datasets with all the known protein coding genes of human (based on Ensembl gene annotations, GRCh38, version 94). In details, for each of the genes with alternative splicing, one of the isoforms was randomly selected as “highly expressed” and the other ones were selected as “lowly expressed”. And for the genes with single isoforms, all their isoforms were selected as “highly expressed”. Then high (30X) and low (4X) coverage simulations were implemented by the simulator (PBSim or NanoSim) for the highly and the lowly expressed isoforms respectively, and the simulated reads were mixed to build the dataset for benchmark.*

*We have updated current manuscript as follows:*

*“For each model, there were 10 simulated datasets from three species based on Ensembl gene annotations [38] (human: GRCh38, version 94; mouse: GRCm38, version 94; fruit fly: BDGP6, version 94). One was generated by all coding genes of human to mimic the variable isoform level expressions. In details, for each of the genes with multiple isoforms, one of the isoforms was randomly selected as “highly expressed” and the other ones were selected as “lowly expressed”. And for the genes with single isoforms, all their isoforms were selected as “highly expressed”. Then high (30X) and low (4X) coverage simulations were implemented for the highly and the lowly expressed isoforms, respectively, and the simulated reads were mixed to build the dataset.” (line 39, page 6 of main text)*

*“H-all transcriptome was composed by all the coding genes recorded in Ensembl gene annotations of human. For each of the genes with alternative splicing, one of the isoforms was randomly selected as “highly expressed” and the other isoforms were “lowly expressed”, and all the genes with single isoform were “highly expressed”. Then transcript sequences were made for all the isoforms of all the genes, i.e., for a specific gene isoform, all its exons were concatenated according to the Ensembl annotations to build a transcript sequence.*

*Given a sequencing model, the sequences of highly and lowly expressed isoforms were input to the specifically configured simulator (PBSim or NanoSim) to implement a 30X and a 4X coverage in silico sequencing, respectively, and the two generated datasets were mixed as one for the use of benchmark. Thus, there were in total 6 datasets simulated with H-all transcriptome by various sequencing models. Herein, we used the term “coverage” only as a measure of the sizes of the datasets. That means, a specific sequencing coverage dX (e.g., d = 30) indicates that a set of transcript sequences were input into the simulator to produce a dataset whose total size is L_TS×d, where L_TS is the total length of the transcript sequences.” (line 36, page 16 of main text)*

*Overall, the yields of deSALT on these newly added datasets are still better than those of other aligners, and the difference between the alignments of the reads from highly- and lowly expressed isoforms is quite small. This indicates that deSALT enables to handle the reads from lowly expressed isoforms. Please refer to Supplementary Figure 4 and Supplementary Tables 5 and 6 for the results, and an example is shown in Supplementary Figure 3.*

*It is also observed that, on high error rate datasets (e.g., ONT 1D reads), the yields of the reads from lowly expressed isoforms are a little bit lower than that of highly expressed isoforms. We further asked deSALT to separately align the reads from lowly- and highly expressed isoforms (as two independent datasets), and similar results were observed for the reads from lowly expressed isoforms. Thus, we realized that the difference between the yields of lowly- and highly expressed isoforms were not caused by the variable isoform level expressions, but the absolute numbers of the reads. That means, there are few reads from lowly expressed isoforms and they are not enough to mitigate the serious sequencing errors with the two-pass approach of deSALT. This is quite like the alignment of low coverage high error rate datasets (e.g., the simulated 4X ONT 1D datasets). If the total sequencing coverage increases, i.e., in absolute term there are more reads sequenced from the lowly expressed isoforms, the alignment of such reads would be as good as that of highly expressed isoforms, regardless of how many reads being sequenced from other isoforms.*

*We have updated current manuscript as follows:*

*“To further investigate the ability of the aligners on the reads from alternative splicing genes, we assessed the alignments of the reads from “highly expressed isoforms” and “lowly expressed isoforms” of all human coding genes datasets (Supplementary Table 6). The yields of deSALT were still better than those of other aligners, and the difference between the reads from highly- and lowly expressed isoforms was overall quite small. However, for error-prone ONT 1D reads, the difference between lowly- and highly expressed isoforms was larger, e.g., there was a 10% decrease for the exon% statistics (Supplementary Figure 4). We investigated some intermediate results of deSALT, and found that the decreased statistics are not due to a poor ability of handling alternative splicing genes, but the low coverage of the datasets, which is similar to that of the low coverage ONT 1D datasets. That is, there were only a small number of reads being from lowly expressed isoforms, and they were not enough to mitigate the sequencing noise with their own alignment skeletons. Thus, some difficult and isoform-specific cases could not be well handled, e.g., some read parts from isoform-specific short exons. To further justify this issue, we used the ONT 1D reads from lowly expressed isoforms as an independent dataset and asked deSALT to run it. Similar results were obtained (the red bars in Supplementary Figure 4), and this suggested that deSALT was not affected by the variable expression levels of the isoforms.” (line 58, page 8 of main text)*

- How does deSALT perform on pairs of genes that overlap genomically but are on opposite strands? Would the potential for overlapping exons cause problems for alignment accuracy? Additional tests highlighting this issue are needed.

*--Response:*

*deSALT is also able to handle the reads from pairs of genes that overlap genomically but are on opposite strands, and the overlapped exons would not cause problems for alignment accuracy. This derives from the implementations of deSALT that it considers the various strands during exon inference and refined alignment. Moreover, in the refined alignment step, it uses a re-alignment strategy to correct LSRS to better handle alternative splicing sites. We have added a benchmark on the reads from the overlapped genes to explain this issue. The results are given in Supplementary Figure 5 and Supplementary Table 7, and an example has also been added in Supplementary Figure 6.*

*We have added the description in the manuscript:*

*“Moreover, there is also a category of special cases that pairs of genes overlap in the genome but are on opposite strands. The alignment of the reads from such genes are to some extent similar to that of the reads from alternative splicing genes. We implemented an assessment on the alignments of these reads (Supplementary Table 7), and found that there was no significant difference to that of deSALT’s alignment results on the reads from non-overlapped genes (Supplementary Figure 5), indicating that deSALT can also produce accurate alignments for such reads. An example is in Supplementary Figure 6.” (line 27, page 9 of main text)*

- What is the memory use of all the methods?

*--Response:*

*We have added the memory footprints of the benchmarked aligners in Figure 2C and Supplementary Table 8. We have added some sentences to describe this issue.*

*We mentioned in the current version of the manuscript as follows:*

*“Moreover, the aligners have different memory footprints (Figure 2C and Supplementary Table 8). The memory footprints of deSALT is higher than that of Minimap2 and GMAP, since the RdBG-index of deSALT uses a hash table-based data structure to index all the k-mers of RdBG, which needs larger RAM space. However, the memory use of deSALT is still acceptable to most of modern servers (e.g., about 35GB for human). It is also observed that the memory footprints of GraphMap2 increased with the number of input reads, and higher than that of all the other benchmarked aligners.” (line 6, page 8 of main text)*

- What are the memory/time footprint of the indexing stage? I tested deSALT indexing on GRCh38 and it took over 70Gb and over two hours.

*--Response:*

*Thank you for pointing this out. We have added the memory footprints of the construction of RdBG-index.*

*We have added the description in the manuscript:*

*“It is worth noting that, the construction of RdBG-index for large genomes could cost a couple of hours (129, 112 and 4 minutes for human, mouse and fruit fly, respectively) and several tens of GB RAM space (73GB, 63GB and 5.5GB for human, mouse and fruit fly, respectively), depending on the number of distinct k-mers in the genome. This is mainly because that it needs to extract and sort all the k-mers to construct RdBG and analyze its unitigs at first. However, it is also affordable since the index needs only to be built once before use, and we also provide pre-built RdBG-indexes for human, mouse and fruit fly reference genomes (Supplementary Notes).” (line 38, page 13 of main text)*

- Since GMAP and deSALT prebuild their indices, when comparing to Minimap2 its index should also be prebuilt. The command provided does not seem to indicate that.

*--Response:*

*Thank you for pointing this out. The index of Minimap2 was also pre-built in the benchmark (for both of simulated and real datasets). We have added description in the main text to clarify this issue:*

*“deSALT and three state-of-the-art approaches, Minimap2, GraphMap2 and GMAP, were applied to all 60 datasets for comparison. In the benchmark, the indexes of all the aligners were pre-built.” (line 1, page 7 of main text)*

*Moreover, the command lines of the index and alignment of Minimap2 are given in the Supplementary Notes.*

*We have mentioned this in the current version of the manuscript:*

*“7.2 Command lines of Minimap2 (version 2.17-r941)*

*Genome indexing:*

*minimap2 -ax splice -d ref.mmi ref.fa*

*Read alignment*

*minimap2 -ax splice -t 24 ref.mmi Fastq_File > aln.sam” (line 31, page 143 of Supplementary Notes)*

\#### Methods

- It is not clear how the RdBG index is queried with l-mers given that the index was constructed with k-mers of size k>l. What is the auxiliary data structure that allows that?

*--Response:*

*Thank you for pointing this out. RdBG-index allows to retrieve the k-mers whose initial l characters (i.e., the l long prefix) are same to a given l-mer. This is implemented by the data structures of RdBG-index and a specifically designed method. We have added some new paragraphs in Supplementary Notes and a new schematic illustration (Supplementary Figure 12) to more precisely explain the data structures used by RdBG-index and the methods to query l-mers (lWe have updated the manuscript as follows:*

*“In details, RdBG-index has four major data structures as follows (a schematic illustration is in Supplementary Figure 12).*

*1) A linear table (UT_seq) records the sequences and the IDs (called as U_ID) of all the unitigs of RdBG, each unitigs has a unique U_ID.*

*2) A linear table (UT_kmer) records the lexicographically sorted list of all the k-mers of RdBG. For each of the k-mers, UT_kmer records its token and its unique position in RdBG, i.e., the U_ID and the offset of the unitig the k-mer being at.*

*3) A hash table (UT_k') records the ranges of the k-mers having identical initial k’ charaters in UT_kmer. UT_k' is straightforwardly implemented by a surjection of k’-mers to RAM space, i.e., UT_k' records all the 4^k’ k’-mers in a lexicographical order in the RAM, and each item of UT_k' records a specific range of the k-mers of RdBG whose k’ long prefixes (also called as k’-prefixes) are same to the corresponding k’-mer. It is worth noting that for some items their ranges could be null, due to that there is no such k-mers in RdBG. This is an auxiliary data structure which helps to find short token matches between a read and the reference. As a sorted list, binary search is needed to locate a specific k-mer in UT_kmer, which is quite time consuming. UT_k' helps to narrow down the range of k-mer retrieval and accelerate the query. The configuration of parameter k’ is critical and deSALT sets k’ = 14 (same as that of deBGA) which is a tradeoff between speed and memory use. Thus, the size of UT_k' is 2GB, i.e., there are 4^14 items and each of them stores one integer to record the range. Herein, the ranges are recorded in a difference approach, so that only one integer is needed to record in an item, and a range can be derived from two neighboring items.*

*4) A linear table (UT_pos) records the genomic positions of the untigis, i.e., for each of the unitigs, the starting positions of all its copies in the reference are recorded. UT_pos enables to convert the matches on unitigs to the matches on reference.” (line 8, page 136 of Supplementary Notes)*

*“It is worth noting that, RdBG-index not only supports to find k-mer matches, but also supports to find the matches from a l-mer (l*

- "Implementation of the simulation benchmark":

- How was the coverage calculated? Since we are dealing with transcriptome here the definition of coverage is not as clear it is in the case of genomics.

*--Response:*

*In the manuscript, we used the term “coverage” only as a measure of the sizes of the simulated datasets. A specific sequencing coverage dX (e.g., d = 30) indicates that a set of transcript sequences were input into the simulator to produce a dataset whose total size is L_TS×d, where L_TS is the total length of the transcript sequences. We have revised the sentences to clarify this issue:*

*“Herein, we used the term “coverage” only as a measure of the sizes of the datasets. That means, a specific sequencing coverage dX (e.g., d = 30) indicates that a set of transcript sequences were input into the simulator to produce a dataset whose total size is L_TS×d, where L_TS is the total length of the transcript sequences.” (line 54, page 16 of main text)*

- The second step is not clear. Specifically, the second sentence: "the transcript sequences were generated according to all its isoforms"

*--Response:*

*Thank you for pointing this out. Each simulated dataset was produced based on a specific set of gene isoforms, and for each of the gene isoforms, a transcript sequence was made by concatenating all its exons according to the gene annotations. We also realized that the paragraphs of “Implementation of the simulation benchmark” were not clearly written, meanwhile, we also added new simulated datasets in the current version of the manuscript as well. So, we revised the paragraphs about the data simulation method to improve the readability:*

*“All the benchmarks were implemented on a server with Intel Xeon E4280 CPU at 2.0GHZ and 1 Terabytes RAM, running Linux Ubuntu 16.04. The simulated datasets were generated from the reference of three organisms: Homo sapiens GRCh38 (human), Mus musculus GRCm38 (mouse), and Drosophila melanogaster r6 (fruit fly), with corresponding Ensembl gene annotations [38]. There are 60 datasets used for the benchmark, and each of them was generated by a specific combination of sequencing model, simulated transcriptome and coverage.*

*6 sequencing models were built according to previous studies [17, 35, 36], to comprehensively benchmark the aligners on the datasets produced by various long read sequencing platforms. For PacBio platforms, there were two models built with fixed parameters: “PacBio ROI reads” (error rate = 2%, mean read length = 2000 bp) and “PacBio subreads” (error rate = 15%, mean read length = 8000 bp). For ONT platforms, four models were built by which two of them were also with fix parameters: “ONT 2D reads” (error rate = 13%, mean read length = 7800 bp) and “ONT 1D reads” (error rate = 25%, mean read length = 7800 bp). And the other two models, “PS-ONT reads” and “NS-ONT reads” were automatically built by PBSim [37] and NanoSim [36] based on a real ONT sequencing dataset (SRA Accession Number: SRR2848544), respectively. Although previous studies [36] indicate that data-based models more coincide with the characteristics of real sequencing, we considered that real ONT datasets could also have various characteristics [35] and it is hard to build a number of models for benchmark. Thus, we used the two parameter-based models, “ONT 2D reads” and “ONT 1D reads”, as a complement, where the 25% and 12% error rates coincide with typical error rates of ONT 2D (1D2) and 1D reads [18, 19]. The parameters and command lines of PBSim and NanoSim are in Supplementary Notes.*

*We built two categories of simulated transcriptomes. The first category only has one simulated transcriptome (called as “H-all” transcriptome) which is from all the coding genes of human, and the second category has three simulated transcriptomes (respectively called as “H-se”, “M-se” and “F-se” transcriptomes) which are from three sets of randomly selected genes of human, mouse and fruit fly, respectively. Each of the transcriptomes was used to generate a series of simulated datasets with various sequencing models. Some details are as follows:*

*H-all transcriptome was composed by all the coding genes recorded in Ensembl gene annotations of human. For each gene with alternative splicing, one of the isoforms was randomly selected as “highly expressed” and other isoforms were as “lowly expressed”, and all the genes with single isoform were as “highly expressed”. Then transcript sequences were made for all the isoforms of all the genes, i.e., for a specific gene isoform, all its exons were concatenated according to the Ensembl annotations to build a transcript sequence.*

*Given a sequencing model, the sequences of highly and lowly expressed isoforms were input to the specifically configured simulator (PBSim or NanoSim) to implement a 30X and a 4X coverage in silico sequencing respectively, and the two generated datasets were mixed as one for the use of benchmark. Thus, there were in total 6 datasets simulated with H-all transcriptome by various sequencing models. Herein, we used the term “coverage” only as a measure of the sizes of the datasets. That means, a specific sequencing coverage dX (e.g., d = 30) indicates that a set of transcript sequences were input into the simulator to produce a dataset whose total size is L_TS×d, where L_TS is the total length of the transcript sequences.*

*H-se, M-se and F-se transcriptomes were composed in a similar way to a previous study [30]. For a specific species, the gene annotations were scanned to extract three sets of genes. Each of them corresponds to a specific type, i.e., genes with single splicing isoforms, genes with multiple splicing isoforms, and genes with short exons (<31 bp), respectively. A number of genes were randomly selected for each of the gene sets and other genes were no longer used (refer to Supplementary Table 11 for the numbers of selected genes). Transcript sequences were then made for the selected genes with the same approach, i.e., given a gene isoform, a transcript sequence was made by concatenating all its exons, and the sequences were made for all the isoforms of all the selected genes. H-se, M-se and F-se transcriptomes were then composed by the generated transcript sequences of human, mouse and fruit fly, respectively*

*For each of the transcriptomes (H-se, M-se or F-se), 3 datasets with various coverages, i.e., 4X, 10X and 30X were simulated by a given sequencing model. Thus, with the 3 transcriptomes, 3 kinds of coverages and 6 models, there were in total 54 datasets produced. These datasets are used to assess the ability of the aligners on various species, coverage and platforms.” (line 48, page 15 of main text)*

- How were the specific genes in each of the three gene sets selected? Were they randomly selected? If there is no strong reason then I think that all protein coding genes should be included in their respective gene sets?

*--Response:*

*Thank you very much for this good suggestion. We referred to a previous study proposed by Križanovic et al. [1] to use several sets of randomly selected genes to implement data simulation. Mainly, the three gene sets were randomly selected with Ensembl gene annotations. We used an in-house program to scan gene annotation files to collect three sets of genes. Each of them corresponds to a specific type, i.e., genes with single splicing isoforms, genes with multiple splicing isoforms, or genes with short exons (<31 bp), respectively. A number of genes were randomly selected from each of the gene sets and other genes were no longer used. In the benchmark, the numbers of selected genes are about two times higher than that of the largest dataset used in Križanovic et al.’s study. We have revised the corresponding sentences to make it clearer:*

*“For a specific species, the gene annotations were scanned to extract three sets of genes. Each of them corresponds to a specific type, i.e., genes with single splicing isoforms, genes with multiple splicing isoforms, and genes with short exons (<31 bp), respectively. A number of genes were randomly selected for each of the gene sets and other genes were no longer used (refer to Supplementary Table 11 for the numbers of selected genes). Transcript sequences were then made for the selected genes with the same approach, i.e., given a gene isoform, a transcript sequence was made by concatenating all its exons, and the sequences were made for all the isoforms of all the selected genes. H-se, M-se and F-se transcriptomes were then composed by the generated transcript sequences of human, mouse and fruit fly, respectively.” (line 1, page 17 of main text)*

*We did not use all the protein coding genes in the previous version of the manuscript, mainly because that the sizes of the datasets would be very large (several times larger than the used datasets) if all of them were used. As there are quite a lot of datasets with various sequencing models, coverages and organisms (i.e., 36 datasets for previous version of the manuscript, and 60 datasets for the current version of the manuscript), and all the benchmarked aligners (deSALT, deSALT+annotation, Minimap2, Minimap2+annotation, GMAP and GraphMap2) need to run, it will cost a lot of time to implement the benchmark with limited computational resources. Especially, some of the aligners have relatively slow speed, e.g., GMAP cost more than 30 days to run all the simulated and real datasets with one server, even if multiple threads were used. Under such circumstance, the time cost of the benchmark could be to some extent prohibitive, if all the protein coding genes are used to simulate the datasets by the same approach.*

*We agree with the reviewer that it is helpful to comprehensively benchmark the aligners with all the protein coding genes. In the current version of the manuscript, we have added 6 new simulated human datasets, which include all the protein coding genes of human. Moreover, in these datasets, for each of the genes with multiple splicing isoforms, one of the isoforms had a higher sequencing coverage (30X) and other isoforms had lower sequencing coverage (4X), to mimic the variable expression levels of the multiple isoforms of the same gene. We believe that these datasets are helpful to more comprehensively benchmark the aligners. Similar trends have been observed from the results of the aligners on these datasets. Please refer to Figure 2 and Supplementary Tables 2-8 for the results.*

*We have mentioned this in the current version of the manuscript:*

*“We built two categories of simulated transcriptomes. The first category only has one simulated transcriptome (called as “H-all” transcriptome) which is from all the coding genes of human, and the second category has three simulated transcriptomes (respectively called as “H-se”, “M-se” and “F-se” transcriptomes) which are from three sets of randomly selected genes of human, mouse and fruit fly, respectively. Each of the transcriptomes was used to generate a series of simulated datasets with various sequencing models. Some details are as following.*

*H-all transcriptome was composed by all the coding genes recorded in Ensembl gene annotations of human. For each gene with alternative splicing, one of the isoforms was randomly selected as “highly expressed” and the other isoforms were “lowly expressed”, and all the genes with single isoform were “highly expressed”. Then transcript sequences were made for all the isoforms of all the genes, i.e., for a specific gene isoform, all its exons were concatenated according to the Ensembl annotations to build a transcript sequence.*

*Given a sequencing model, the sequences of highly and lowly expressed isoforms were input to the specifically configured simulator (PBSim or NanoSim) to implement a 30X and a 4X coverage in silico sequencing respectively, and the two generated datasets were mixed as one for the use of benchmark. Thus, there were in total 6 datasets simulated with H-all transcriptome by various sequencing models. Herein, we used the term “coverage” only as a measure of the sizes of the datasets. That means, a specific sequencing coverage dX (e.g., d = 30) indicates that a set of transcript sequences were input into the simulator to produce a dataset whose total size is L_TS×d, where L_TS is the total length of the transcript sequences.” (line 25, page 16 of main text)*

- Where are GMAP results in Figure 2 for ONT 1D data?

*--Response:*

*Thank you for pointing this out. In this plot, the bars corresponding the results of GMAP are unclear because the speed of GMAP is quite slow on ONT 1D datasets. We have added extra data labels in the current version of the figure (Page 25 of main text), to more clearly show the results of GMAP as well as other aligners.*

- "3.2 Alignment of the read part against the LSRS":

- What constitutes "large deletions" in the CIGAR?

*--Response:*

*Most of the large deletions in the CIGAR are caused by the exons having alternative splicing sites. For such exons, their whole sequences are inferred by deSALT in the exon inference step and composed into LSRSs in the refined alignment step. However, the exon sequences could be partially included in some gene isoforms due to alternative splicing sites, so that the reads from those transcripts only have partial sequences. In this situation, there could be large deletions when the reads are aligned against the LSRSs involving the exons having alternative splicing sites. deSALT checks such deletions in the CIGARs, and removes the bases according to the large deletions to update the LSRSs and realign the reads. This helps to improve the accuracy of the alignment. We have revised some sentences and added a schematic illustration (Supplementary Figure 13) to describe this point more clearly:*

*“For each of the alignments, the CIGAR string is investigated. If there are large deletions, deSALT updates the LSRS by removing the bases corresponding to the large deletion(s) and realigns the read part. Herein, most of the large deletions are due to the exons having alternative splicing sites. For such exons, their whole sequences are inferred by deSALT in the exon inference step and composed into LSRSs. However, the exon sequences could be partially included in some gene isoforms due to their alternative splicing sites, so the reads from those transcripts only have partial sequences. In this situation, there could be large deletions when the reads are aligned against the LSRSs involving the exons having alternative splicing sites. A schematic illustration is in Supplementary Figure 13.” (line 5, page 141 of Supplementary Notes)*

- "2.2 Exon refinement":

- How are the accepter/donor matrices derived?

*--Response:*

*Two scoring matrixes—Acceptor Scoring Matrix and Donor Scoring Matrix are used by deSALT to refine the inferred exon boundaries to obtain more accurate exon regions. These two scoring matrixes were proposed by Kim et al.’s previous study of splice site identification [2]. We have added the citation in Supplementary Notes and the main text. A brief sentence describes this issue as follows:*

*“……Acceptor Scoring Matrix and Donor Scoring Matrix (see below), to respectively score the positions in EA_p and ED_p. The two matrixes were initially proposed by Kim et al. to identity splice sites and translation start sites in human genomic sequences [5].” (line 28, page 139 of Supplementary Notes)*

*“[5] Kim K-B, Park K, Kong EBJJob, biology m: A method for identifying splice sites and translation start sites in human genomic sequences. 2002, 35:513-517.” (line 15, page 144 of Supplementary Notes)*

\### Minor Comments

- For ease of read, I suggest converting all time into minutes and all memory to GB.

*--Response:*

*Thanks for the suggestion and we have converted all the runtimes into minutes and all the memory footprints to GB. Please refer to Supplementary Tables 8 and 9.*

- "Results on simulated datasets":

- What is meant by "the Exon% statistics (about 64%-90%) were affected to some extent"? What is the percentage range referring to?

*--Response:*

*The percentage range 64%-90% indicates the exon% statistics of deSALT on the simulated ONT 1D datasets. We have revised the sentence to describe it more clearly.*

*We have revised the manuscript as follows:*

*“But for the error-prone ONT 1D reads (error rate: 25%), the exon% statistics of deSALT is about 64%–90%, lower than that of other simulated datasets. This indicates that deSALT was to some extent affected by the high sequencing errors.” (line 34, page 7 of main text)*

- P4L9: "most of them are relatively low speed" --> "most of them have relatively slow speed"

Collapse

*--Response:*

*Thank you for pointing this out. We have corrected the sentence:*

*“However, most of them have relatively slow speed [28, 29], mainly because of the numerous short matches in the seeding step and the time-consuming local alignment in the extension step.” (line 11, page 4 of main text)*

- (not required but highly recommended) Please consider adding the deSALT to bioconda packages for making it more accessible for general audience.

*--Response:*

*Thank you for the suggestion. We have added deSALT to bioconda packages. Users can install deSALT by the following command line:*

*conda install -c bioconda desalt*

---------------------------------------------------------------------------------------------------------------

Response to reviewer #2

---------------------------------------------------------------------------------------------------------------

Reviewer #2: In this manuscript, Liu et al. introduce deSALT, a new method of aligning long-read transcriptomic data against a reference genome using a de Bruijn graph-based index. This approach builds upon some of the previous work of some of the authors (the RdBG index, as introduced in deBGA), but makes entirely different use of this index and also adds many novel concepts specifically related to the alignment of long spliced (i.e. transcriptomic) reads. deSALT looks like a very promising new approach, and the manuscript provides a detailed and precise description of the method. The assessment is generally convincing. However, there are some places where extra information could be provided or the manuscript might be improved.

*--Response:*

*Thanks for the comments and suggestions of the reviewer. We have supplied new benchmark results and revised the manuscript according to the reviewer’s comments. A point-by-point response has been made according to the comments as follows.*

* It seems that an assessment of the memory requirements is not given anywhere within the current manuscript. Along with accuracy and alignment speed, the memory usage of the proposed approach is a critical metric to report, as it informs in which contexts the tool might be used (e.g. on cluster nodes with limited memory, or in memory-limited cloud instances).

*--Response:*

*We have added the memory footprints of the benchmarked aligners in Figure 2C and Supplementary Table 8. The memory footprint of deSALT is higher than that of Minimap2 and GMAP, but lower than GraphMap2. We also added some sentences to describe it.*

*We have revised the manuscript as follows:*

*“Moreover, the aligners have different memory footprints (Figure 2C and Supplementary Table 8). The memory footprints of deSALT is higher than that of Minimap2 and GMAP, since the RdBG-index of deSALT uses a hash table-based data structure to index all the k-mers of RdBG, which needs larger RAM space. However, the memory use of deSALT is still acceptable to most of modern servers (e.g., about 35GB for human). It is observed that the memory footprints of GraphMap2 increased with the number of input reads, and higher than that of all the other benchmarked aligners.” (line 6, page 8 of main text)*

* The authors state that PBSim is used for generating all of the simulated data, where different types of simulated samples are created by changing the characteristic read lengths and error rates. However, the error characteristics of PacBio and ONT data differ in many important ways apart from simply the rate of error. Thus, it is unlikely that reads generated by PBSim with a high error rate and a longer mean read length are truly representative of how nanopore reads are likely to look. I'd recommend that the authors use a nanopore-specific simulator to benchmark on simulated ONT data --- there are a few options available like NanoSim or DeepSimulator.

*--Response:*

*Thank you very much for this helpful suggestion. We agree that NanoSim and DeepSimulator enable to simulate more realistic ONT datasets with tailored machine learning-based approaches, and this is helpful to benchmark the aligners more comprehensively. So, we added 10 new datasets simulated by NanoSim. A real ONT dataset (SRA Accession Number: SRR2848544) was employed for the simulation as required by NanoSim. Meanwhile, we were thinking that PBSim can also generate sequencing profiles from real datasets to simulate more realistic datasets, so we used the same real ONT dataset to simulate 10 new datasets by PBSim, and employed them in the benchmark as well. Thus, 20 new datasets produced by the two simulators based on real ONT data have been added in the current version of the manuscript. The results (Figure 2 and Supplementary Tables 2-8) suggested that the trend is similar to previously used datasets.*

*Moreover, we considered that the characteristics of various real ONT datasets could be different, but it is very hard to simulate many datasets with a number of real ONT datasets. So, we kept the parameter-based simulated ONT datasets (i.e., the simulated ONT 1D and ONT 2D reads) in the benchmark as a reference, since their error rates are similar to typical real ONT 1D and ONT 2D sequencing reads.*

*We have revised the manuscript as follows:*

*“For ONT platforms, four models were built. Two of them were also with fix parameters: “ONT 2D reads” (error rate = 13%, mean read length = 7800 bp) and “ONT 1D reads” (error rate = 25%, mean read length = 7800 bp). And the other two models, “PS-ONT reads” and “NS-ONT reads” were automatically built by PBSim [37] and NanoSim [36] based on a real ONT sequencing dataset (SRA Accession Number: SRR2848544), respectively. Although previous studies [36] indicate that data-based models more coincide with the characteristics of real sequencing, we considered that real ONT datasets could also have various characteristics [35] and it is hard to build a number of models for benchmark. Thus, we used the two parameter-based models, “ONT 2D reads” and “ONT 1D reads”, as a complement, where the 25% and 12% error rates coincide with typical error rates of ONT 2D (1D2) and 1D reads [18, 19]. The parameters and command lines of PBSim and NanoSim are in Supplementary Notes.” (line 5, page 16 of main text)*

* When performing an assessment on experimental samples, it would be useful to look at both cDNA and direct RNA-based nanopore sequencing. Though similar, the characteristics of these protocols are not identical, and it seems important to know how the different methods perform on both types of data (though I have no a priori reason to believe that deSALT's improvements won't hold equally-well in both situations).

*--Response:*

*Thank you for this helpful suggestion. Adding real direct RNA sequencing dataset is helpful to implement a more comprehensive benchmark. We have added the direct RNA dataset from NA12878 sample, and the results are shown in Figure 4 and Supplementary Tables 9 and 10. As expected, the trend is similar to that of cDNA datasets.*

*We have revised the manuscript as follows:*

*“We assessed the aligners with three real sequencing datasets. The first two datasets are from a well-studied CEPH sample (NA12878), and respectively produced by ONT cDNA sequencing (containing 15,152,101 reads and 14,134,831,170 bases in total) and ONT direct RNA sequencing (containing 10,302,647 reads and 10,614,186,428 bases in total). The two datasets are available at https://github.com/nanopore-wgs-consortium/NA12878. The third dataset is from a mouse sample produced by the PacBio platform [39] (SRA Accession Number: SRR6238555; containing 2,269,795 reads and 3,213,849,871 bases in total).” (line 8, page 10 of main text)*

*Please also refer to “Results on real sequencing datasets” section for more detailed information about the results on the direct RNA dataset.*

* Given that deSALT builds a secondary k'-mer index (where k' < k) index over the RdBG index, it was not particularly clear what the benefit or motivation was for using a graph-based index as opposed to simply building a k'-mer index over the input reference. When k' = k (as in deBGA), there is a benefit that any U-MEM is unique within the dBG, however in the case k' < k, only U-MEMS of length at least k are guaranteed to be unique in the dBG. Is it the case the performing the initial seeding over the dBG as opposed to the reference improves efficiency by reducing redundancy during seeding? Is this still the case even though k' < k? The authors state that altering the value of k has little effect on the results or the runtime, but what is the effect of changing the value of k'. This is a crucial parameter for this algorithm, and so some assessment of its effect would be useful to have.

*--Response:*

*deSALT uses l-mers (lWe have revised the manuscript as follows:*

*“deSALT further merges co-linear U-MEMs on same unitigs as super U-MEMs (SU-MEMs). This is implemented by the following rule: for two U-MEMs M_1and M_2 on the same unitig ux, M_1=(M_1^RS,M_1^RE,M_ux^U,M_1^US,M_1^UE ) and M_2=(M_2^RS,M_2^RE,M_ux^U,M_2^US,M_2^UE ), if |(M_2^RS-M_1^RS )-(M_2^US-M_1^US )|The sequencing error is a critical point to the configuration of seed length, i.e., the l parameter. For the noisy long reads, l should not be set too large as there are few long exact matches. We referred to some state-of-the-art long read aligners, such as Minimap2, BLASR and BWA-MEM, and also did some testing on simulated datasets to configure l parameter. We have added some of the testing results in the current version of the manuscript. Please refer to Supplementary Table 12.*

*We have revised the manuscript as follows:*

*“For noisy long reads, l should not be set too large otherwise there could be few long exact matches. To investigate its effects, we configured l parameter with various settings (l = 14, 15, 17 and 19), and implemented deSALT on the 18 low-coverage (4X) simulated datasets from human, mouse and fruit fly (with the 6 simulation models). The results are in Supplementary Table 12. Considering the alignment yields, we selected the default value of l as 15 and used this configuration in all the benchmarks, except for simulated ONT 1D reads, which l=14 was used due to the high sequencing errors.” (line 8, page 137 of Supplementary Notes)*

*Moreover, we think that there could be a little misunderstanding about the secondary k'-mer index of RdBG-index data structure. There is such an auxiliary data structure (termed as UT_k' in RdBG-index) and its function is to accelerate the query of RdBG-index. However, the seed length, i.e., parameter l, does not directly relate to the implementation of the secondary k'-mer index. In fact, there are specifically designed approaches to find matches between l-mers and the k-mers indexed by RdBG-index.*

*We considered the tradeoff between speed and memory footprint, and set the k’ parameter as 14 for the secondary k'-mer index. With this configuration, deSALT can achieve a fast speed with an acceptable memory footprint. In the current version of deSALT, this setting is not allowed to change, but it is still a future work for us to tune this parameter and improve the implementation of deSALT.*

*To explain the implementation and function of RdBG-index more precisely, especially the secondary k'-mer index and the query of l-mers, we added some new paragraphs in “1. Genome indexing” of Supplementary Notes and a new schematic illustration (Supplementary Figure 12). Some details about the data structures used in RdBG are given there.*

*We have revised the manuscript as follows:*

*“In details, RdBG-index has four major data structures as following (a schematic illustration is in Supplementary Figure 12).*

*1) A linear table (UT_seq) records the sequences and the IDs (called as U_ID) of all the unitigs of RdBG, each unitigs has a unique U_ID.*

*2) A linear table (UT_kmer) records the lexicographically sorted list of all the k-mers of RdBG. For each of the k-mers, UT_kmer records its token and its unique position in RdBG, i.e., the U_ID and the offset of the unitig the k-mer being at.*

*3) A hash table (UT_k') records the ranges of the k-mers having identical initial k’ charaters in UT_kmer. UT_k' is straightforwardly implemented by a surjection of k’-mers to RAM space, i.e., UT_k' records all the 4^k’ k’-mers in a lexicographical order in the RAM, and each item of UT_k' records a specific range of the k-mers of RdBG whose k’ long prefixes (also called as k’-prefixes) are same to the corresponding k’-mer. It is worthnoting that for some items their ranges could be null, due to that there is no such k-mers in RdBG. This is an auxiliary data structure which helps to find short token matches between a read and the reference. As a sorted list, binary search is needed to locate a specifc k-mer in UT_kmer, which is quite time consuming. UT_k' helps to narrow down the range of k-mer retrieval and accelerate the query. The configuration of parameter k’ is critical and deSALT sets k’ = 14 (same as that of deBGA) which is a tradeoff between speed and memory use. Thus, the size of UT_k' is 2GB, i.e., there are 4^14 items and each of them stores one integer to record the range. Herein, the ranges are recorded in a difference approach, so that only one integer is needed to record in an item, and a range can be derived from two neighboring items.*

*4) A linear table (UT_pos) records the genomic positions of the untigis, i.e., for each of the unitigs, the starting positions of all its copies in the reference are recorded. UT_pos enables to convert the matches on unitigs to the matches on reference.*

*The size of RdBG-index depends on several factors, mainly the numbers and the lengths of unitigs, the number of the k-mers, the size of the k’-mer hash table and the total numbers of the copies of the unitigs. For human (GRCh38, version 94), mouse (GRCm38, version 94) and fruit fly (BDGP6, version 94) reference genomes, the sizes of the index are 35GB, 31GB and 3.5GB, respectively (with paramerter k=22 and k’=14).*

*It is worth noting that, RdBG-index not only supports to find k-mer matches, but also supports to find the matches from a l-mer (l*

* It would be useful if the authors provide some technical detail about the k'-mer index they build over the RdBG. Is this simply a hash table with a list of the positions of each k'-mer in the RdBG? How are the occurrences encoded? What is the size of this data structure and how does it depend on k'? Are the k'-mers canonicalized?

*--Response:*

*Mainly, the k'-mer index is a secondary data structure in RdBG-index, which helps to accelerate the query of short token matches between a read and the reference. It is a hash table-based data structure, which is straightforwardly implemented by a surjection of k’-mers to RAM space, i.e., it records all the 4^k’ k’-mers in a lexicographical order in the RAM (the k'-mers are not canonicalized), and each item of the hash table records a specific range of the k-mers of RdBG whose k’ long prefixes (also called as k’-prefixes) are same to the corresponding k’-mer. Thus, the size of this data structure is O(4^k'), which depends on the parameter k'. In the current version of deSALT, we select k’= 14 by considering the tradeoff between speed and memory use.*

*We have added the new paragraphs in “1. Genome indexing” of Supplementary Notes and a new schematic illustration (Supplementary Figure 12) to more precisely describe the implementation of RdBG-index. The used data structures and the size of RdBG-index, and its relationship to the parameter k’ are given there.*

*We have revised the manuscript as follows:*

*“In details, RdBG-index has four major data structures as follows (a schematic illustration is in Supplementary Figure 12).*

*1) A linear table (UT_seq) records the sequences and the IDs (called as U_ID) of all the unitigs of RdBG, each unitigs has a unique U_ID.*

*2) A linear table (UT_kmer) records the lexicographically sorted list of all the k-mers of RdBG. For each of the k-mers, UT_kmer records its token and its unique position in RdBG, i.e., the U_ID and the offset of the unitig the k-mer being at.*

*3) A hash table (UT_k') records the ranges of the k-mers having identical initial k’ charaters in UT_kmer. UT_k' is straightforwardly implemented by a surjection of k’-mers to RAM space, i.e., UT_k' records all the 4^k’ k’-mers in a lexicographical order in the RAM, and each item of UT_k' records a specific range of the k-mers of RdBG whose k’ long prefixes (also called as k’-prefixes) are same to the corresponding k’-mer. It is worthnoting that for some items their ranges could be null, due to that there is no such k-mers in RdBG. This is an auxiliary data structure which helps to find short token matches between a read and the reference. As a sorted list, binary search is needed to locate a specifc k-mer in UT_kmer, which is quite time consuming. UT_k' helps to narrow down the range of k-mer retrieval and accelerate the query. The configuration of parameter k’ is critical and deSALT sets k’ = 14 (same as that of deBGA) which is a tradeoff between speed and memory use. Thus, the size of UT_k' is 2GB, i.e., there are 4^14 items and each of them stores one integer to record the range. Herein, the ranges are recorded in a difference approach, so that only one integer is needed to record in an item and a range can be derived from two neighboring items.*

*4) A linear table (UT_pos) records the genomic positions of the untigis, i.e., for each of the unitigs, the starting positions of all its copies in the reference are recorded. UT_pos enables to convert the matches on unitigs to the matches on reference.*

*The size of RdBG-index depends on several factors such as the numbers and the lengths of unitigs, the number of the k-mers, the size of the k’-mer hash table and the total numbers of the copies of the unitigs. For human (GRCh38, version 94), mouse (GRCm38, version 94) and fruit fly (BDGP6, version 94) reference genomes, the sizes of the index are 35GB, 31GB and 3.5GB, respectively (with paramerter k=22 and k’=14).” (line 8, page 136 of Supplementary Notes)*

minor comments

-------------------

* There were some places where the phrasing within the manuscript was awkward or value-laden (e.g. "deSALT also obviously outperformed other state-of-the-art aligners"). I would recommend removing such unnecessary adverbs, or adjectives, as they don't really contribute to the point being made.

*--Response:*

*Thank you very much for the suggestion. We have revised the manuscript. For example, the sentence “deSALT also obviously outperformed other state-of-the-art aligners…” has been revised as “deSALT also outperformed other state-of-the-art aligners…” (line 21, page 7 of main text) Other sentences like this have been revised as well in the current version of the manuscript.*

* The formatting of the paper was a bit strange in places. Paragraphs would begin with "#)" followed by a paragraph heading, then a newline before the paragraph begins. I'd suggest the authors make use of more traditional paragraph or subsection headings.

*--Response:*

*In the previous version of the manuscript, we would like to highlight the characteristics of deSALT, but we have realized that the formatting really looked strange. We have used traditional paragraph in the current version of the manuscript.*

* It seems that, if one were to forgo the skeleton creation step, then deSALT may also be appropriate as a DNA-seq aligner for long read data. Have the authors done any testing of the method for this purpose? Can deSALT be used to perform un-spliced alignment of long-read data?

*--Response:*

*Thank you for this valuable suggestion. Generally, deSALT is designed for spliced alignment. Motivated by the frequent splicing events and the sequencing errors of long RNA-seq reads, we realized that it is non-trivial to separately align single reads with high accuracy and sensitivity, so that we implement the two-pass alignment approach. The main idea is to use the information from all the reads (i.e., via the inferred exons) to help all the reads to be better aligned. However, as the reviewer mentioned, most DNA-seq reads need un-spliced alignment. deSALT can construct the alignment skeletons for DNA-seq reads. However, in most of the cases, a single read’s own alignment skeleton is informative enough to produce accurate full-length alignment. (Please also refer to our previous work [3] for this issue.) Under such circumstance, it is not very necessary to use combined alignment skeletons to help the alignment of DNA-seq reads, considering that this could bring extra time cost. Moreover, the combined alignment skeletons of DNA-seq reads may cover the whole reference, so it becomes less informative to later steps. Thus, we do not recommend to use the current version of deSALT for DNA-seq read alignment. We agree with the reviewer that some of the implementations of deSALT are suited to DNA-seq read alignment. It will be an important future work for us to develop novel DNA-seq long read aligner with the techniques of deSALT.*

*Reference*

*[1] Križanovic K, Echchiki A, Roux J, Šikic M: Evaluation of tools for long read RNA-seq splice-aware alignment. Bioinformatics 2018, 34:748-754.*

*[2] Kim K-B, Park K, Kong EBJJob, biology m: A method for identifying splice sites and translation start sites in human genomic sequences. 2002, 35:513-517.*

*[3] Liu B, et al. LAMSA: fast split read alignment with long approximate matches. Bioinformatics 2017, 33(2), 192–201.*

**Second round of review**

**Reviewer 1**

The authors' response to previous comments were satisfactory and comprehensive. One minor comment.

Please include the threshold for what counts as large deletion in the CIGRA.

**Reviewer 2**

I thank the authors for their thorough responses to the comments of both myself and the other reviewer. I congratulate them on the comprehensive manuscript.

**Authors’ response**

Reviewer #1: One minor comment.

Please include the threshold for what counts as large deletion in the CIGRA.

*--Response:*

*deSALT considers the deletions longer than 20 bp in CIGARs as “large deletions”. We have revised the sentence to describe this threshold.*

*We have mentioned in the Supplementary Notes:*

*“If there are large deletions (20 bp is used as a threshold), deSALT updates the LSRS by removing the bases corresponding to the large deletion(s) and realigns the read part.” (line 5, page 141, Supplementary Notes)*

*Thank you very much for reviewing our manuscript.*
